# Unveiling the Hybrid Genome Structure of *Escherichia coli* RR1 (HB101 RecA^+^)

**DOI:** 10.3389/fmicb.2017.00585

**Published:** 2017-04-04

**Authors:** Haeyoung Jeong, Young Mi Sim, Hyun Ju Kim, Sang Jun Lee

**Affiliations:** ^1^Infectious Disease Research Center, Korea Research Institute of Bioscience and BiotechnologyDaejeon, South Korea; ^2^Biosystems and Bioengineering Program, University of Science and TechnologyDaejeon, South Korea; ^3^Korean Bioinformation Center, Korea Research Institute of Bioscience and BiotechnologyDaejeon, South Korea; ^4^Department of Systems Biotechnology, Chung-Ang UniversityAnseong, South Korea

**Keywords:** pedigree, laboratory strain, K-12, evolution, Illumina HiSeq2000

## Abstract

There have been extensive genome sequencing studies for *Escherichia coli* strains, particularly for pathogenic isolates, because fast determination of pathogenic potential and/or drug resistance and their propagation routes is crucial. For laboratory *E. coli* strains, however, genome sequence information is limited except for several well-known strains. We determined the complete genome sequence of laboratory *E. coli* strain RR1 (HB101 RecA^+^), which has long been used as a general cloning host. A hybrid genome sequence of K-12 MG1655 and B BL21(DE3) was constructed based on the initial mapping of Illumina HiSeq reads to each reference, and iterative rounds of read mapping, variant detection, and consensus extraction were carried out. Finally, PCR and Sanger sequencing-based finishing were applied to resolve non-single nucleotide variant regions with aberrant read depths and breakpoints, most of them resulting from prophages and insertion sequence transpositions that are not present in the reference genome sequence. We found that 96.9% of the RR1 genome is derived from K-12, and identified exact crossover junctions between K-12 and B genomic fragments. However, because RR1 has experienced a series of genetic manipulations since branching from the common ancestor, it has a set of mutations different from those found in K-12 MG1655. As well as identifying all known genotypes of RR1 on the basis of genomic context, we found novel mutations. Our results extend current knowledge of the genotype of RR1 and its relatives, and provide insights into the pedigree, genomic background, and physiology of common laboratory strains.

## Introduction

*Escherichia coli* was discovered in 1885 and is the most widely studied organism in molecular biology. It is a versatile model microorganism on which most of the principles and tools of modern genetics and molecular biology are founded (Blount, 2015). Many laboratory strains derived from the wild-type *E. coli* are used in everyday scientific applications as hosts for gene cloning, protein expression, and metabolite production. In addition, *E. coli* includes pathogenic strains that have brought about emerging public health concerns (Kaper et al., 2004; Croxen and Finlay, 2010; Blount, 2015), and is one of the most sequenced species along with other important bacterial pathogens such as *Streptococcus pneumoniae*, *Staphylococcus aureus*, *Salmonella enterica*, and *Mycobacterium tuberculosis*.

Since the complete genome of *E. coli* K-12 (MG1655) was first sequenced in Blattner et al. (1997), it has been regarded as a standard for the study of the K-12 strain, its derivatives, and even (micro)organisms beyond *E. coli*. The continuously updated genome information is available through public online services such as EcoGene (Zhou and Rudd, 2013; Zhou et al., 2013) and EcoCyc (Karp et al., 2014). There is a cautionary note concerning the representativeness of K-12 (Hobman et al., 2007) due to its inherent intraspecies diversity and many genetic changes caused by extended storage in stab culture and/or frequent subculture during its early history. For many decades, a variety of *E. coli* K-12 cells from diverse lineages have been developed for various purposes. The availability of accurate genome information for each strain is crucial to the success of a particular application. Efforts to provide such information include the genome sequencing of *E. coli* W3110 (Hayashi et al., 2006), DH10B (Durfee et al., 2008), BW25113 (Grenier et al., 2014), RV308 (Krempl et al., 2014), TMP32XR1 and TMP32XR2 (Mohan et al., 2015), and MRE600 (Kurylo et al., 2016), and the list of sequenced K-12 strains keeps growing. Even different stocks of the same sequenced strain can harbor genetic variations (Freddolino et al., 2012), which cannot be ignored. The genome sequences of non-K-12 strains, which are used for biotechnological applications (Jeong et al., 2009; Archer et al., 2011), probiotics (Toh et al., 2010; Reister et al., 2014), or phylogenomic studies of *E. coli* (Meier-Kolthoff et al., 2014), are also available.

*Escherichia coli* K-12 RR1 (Bolivar et al., 1977), named after Raymond L. Rodriguez who constructed this strain, is a *recA*^+^ derivative of the HB101 strain (Boyer and Roulland-Dussoix, 1969). RR1 is suitable as a multipurpose cloning host (Bolivar et al., 1977; Maniatis et al., 1982), but it has an advantage over HB101 when a RecA^+^ background is required. Since the first description of RR1 in the literature (Bolivar et al., 1977), many reports mention RR1 as a host for the transformation of pBR322-derived recombinant plasmids (Itakura et al., 1977; Norgard et al., 1979, 1980; Peacock et al., 1981; Imai et al., 1983; Dalrymple et al., 1989). HB101, the parental strain of RR1, is historically important due to its use in elucidating the genetic basis of the host-controlled restriction and modification system in *E. coli* (Loenen, 2003). It is already known that the K-12:B hybrid genome structures of HB101 and RR1 strains come from the transfer of the *hsd-thr* locus of the B strain into the K-12 genomic background.

In this study, we determined the complete genome sequence of RR1 using the Illumina HiSeq platform. Through reference sequence construction, mapping, and revision, an accurate genome sequence was completed with minimal use of PCR and Sanger sequencing. Two recombination junction sites were accurately identified, where the *hsd-thr* genomic sequence and surrounding regions from the B strain were introduced into the K-12 backbone. Based on genome sequence, all known mutations or genotypes in the RR1 strain were also mapped and confirmed. In addition, we discuss how the RR1 strain has evolved and changed in the past based on newly identified mutations.

## Materials and Methods

### Bacterial Strains and Genome Sequencing

*Escherichia coli* strain RR1 (= KCTC 2134 or ATCC 31343) for genome sequencing was purchased from the Korean Collection for Type Cultures (Jeongeup, Jeollabuk-do, South Korea). Cells were grown aerobically in LB medium at 37°C. Genomic DNA was isolated using the Wizard genomic DNA purification kit (Promega, Madison, WI, USA). Library construction using Illumina TruSeq DNA sample preparation kit v2 and 101 cycle paired-end sequencing using the Illumina HiSeq 2000 system were performed according to the manufacturer’s protocol at the National Instrumentation Center for Environment Management (Seoul, South Korea). For PCR-based validation of several mutations of interest, additional RR1 and HB101 cells were purchased from KCTC, Korean Culture Center of Microorganisms (Seoul, South Korea), and TaKaRa Bio (Kusatsu, Shiga, Japan): KCTC 1473 (= ATCC 31343; RR1), KCTC 1467 (= ATCC 33694; HB101), KCCM 70032 (= ATCC 33694; HB101), and HB101 competent cells (TaKaRa cat. No. 9051). Culture condition and DNA isolation method were all the same for the other strains.

### Reconstruction of the Complete Genome Sequence of RR1

Because it is well-known that *E. coli* HB101 and its descendant RR1 had a K-12:B hybrid genome structure, we first constructed a “backbone” genome sequence by *in silico* recombination of K-12 and B genome sequences, where parental regions were determined from the initial read mapping on K-12 MG1655 and BL21(DE3) sequences separately. Subsequent read mapping and sequence correction were carried out, and finally, residual regions that could not be resolved were validated using PCR amplification and Sanger sequencing. Pretreatment of reads (quality limit 0.01, maximum allowed ambiguous base 1, and minimum read length of 50), reference mapping, and subsequent sequence manipulation were carried out using the CLC Genomics Workbench version 6.5.1 (Aarhus, Denmark). Only paired reads passing the pretreatment step were mapped to the reference sequences of *E. coli* K-12 MG1655 (NC_000913.3) and *E. coli* B BL21(DE3) (NC_012971.2) separately, and quality-based variant calling was run. Using putative recombinational junctions inferred from the distribution of single nucleotide variants (SNVs), a hybrid genomic sequence containing part of the B strain genome (190.4 kb) was constructed in the K-12 genomic backbone. Based on this hybrid sequence as the starting reference, a series of sequence manipulation steps consisting of (i) read mapping, (ii) variant detection, and (iii) consensus sequence extraction were iterated until no further mutations were detected. Finally, 25 breakpoints (analyzed by CLC Genomics Workbench) selected on the basis of read number, *p*-values, and fraction non-perfectly mapped, as well as one K-12 island in the B genome background, were grouped in 11 clusters based on genomic location and then corrected using PCR and Sanger sequencing of the amplified products. The Phred/Phrap/Consed package^1^ was used for the final sequence manipulation. Primer sequences and their information are shown in **Table 1**. Amplification targets for primer pairs starting with ‘P’ are shown in **Table 2**. For validation of the *mrr-hsdRMS-mcrBC* deletion, two primer pairs, L-outer:L-inner and R-inner:R-outer, were designed. Because the two inner primers were designed on the basis of the *E. coli* BL21(DE3) genome sequence that does not have deletion, these two primer pairs encompassing deletion junctions would produce amplification products from genomes that have an intact *mrr-hsdR*::IS*1*-*hsdMS-mcrBC* region (967 and 867 bp, respectively). In case of deletion, only the outer primer pair (L-outer:R-outer) would produce a 919-bp product. Primer pairs mlc_F:mcl_R and recA_F:recA_R were used to check mutations in *mlc* and *recA* genes, respectively. Primer pair rhsA-L:rhsA-R and the internal sequencing primer rhsA-I were used for the validation of the *rhsA* sequence. Final validation of the reconstructed RR1 genome sequence was carried out using CLC Genomics Workbench-based re-mapping of Illumina reads and breseq version 0.27.1 (Deatherage and Barrick, 2014). Genome annotation was carried out by the RAST server and NCBI Prokaryotic Genome Annotation Pipeline.

**Table 1:** Primer sequences and their information.

Target location	Product size (bp)	Primer ID and sequence in 5′ to 3′ direction
4,578,424–4,579,228	805	P1 (CAGCGATGGCAGAACA) and P2 (GCTGGCGCACGλT)
4,080,807–4,082,929	2,123	P3 (CCATCAATTTGCTTGGTG) and P4 (GCGCCATTGTTCCTG)
4,323,223–4,325,315	2,093	P5 (TTλATCATCTGCACTTCGTA) and P6 (CCAGCACCTTCλGCAG)
347,834–349,362	1,529	P7 (GCCTGCTCTTATTCTTTCG) and P8 (GGTGCCAACCATTCGG)
2,200,850–2,202,426	1,577	P9 (TCGGTTCATCGAGCATTA) and P10 (CGCGλATTGTGATTATG)
803,901–805,915	2,015	P11 (TGGCGCGTTAACCTTG) and P12 (CCATGCGAGATAATGCCT)
1,547,272–1,549,296	2,015	P13 (CCGCAGCCTCAAGCTC) and P14 (GTCACTCTAATGCGTAATGGA)
1,089,926–1,091,475	1,550	P15 (GCTGCGAATCAGCCAA) and P16 (GCλAGCTGGTCTTCGT)
1,617,797–1,619,902	2,016	P17 (GTλCACGCCCACTCG) and P18 (GCGTTATTGTCGAGTTGATG)
1,942,024–1,942,247	224	P19 (TTTCCTλTCGACGCAAC) and P20 (TGCGCAACATCCCATT)
1,284,742–1,284,976	235	P21 (TTTCCTTAACTGCTTCTCCTC) and P22 (TGCCTTAACλCATCTTTCA)
4,526,800–4,527,718 Δ(*mrr-hsdRMS-mcrBC*)	919	L-outer (CAACACAGGGAGCGAATA) and R-outer (ACAAGATGATGGCGATGG) Inner primers L-inner (TCTGCGTAGTCTTCCTGT) and R-inner (GTTTGCGTTGCGTTTGAG)^a^
2,779,472–2,780,734 (*recA*)	1,272	recA_F (TGTTGATTCTGTCATGGCATATCCTTAC) and recA_R (GCGTATGCATTGCAGACCTTGTGGCAAC)
1,630,858–1,632,327 (*mlc*)	1,450	mlc_F (TCACTAACTCCACCGTTATGCTTC) and mlc_R (GTGCTGTTAATCACATGCCTAAG)
3,718,901–3,721,216 (*rhsA*)	2,316	rhsA-L (GGATGAGλTGAGCGGA) and rhsA-R (ATGCTACCAGAGCAGTGCTT) rhsA-I (TGAGCTTCACCGACTGTT)^b^

^a^*Two inner primers were used to check the intact *mrr-hsdRMS-mcrBC* region as mentioned in the Section “Materials and Methods.”*

^b^*Internal sequencing primer*.

**Table 2:** Large-scale insertions and deletions.

Evidence	Category	Locus	Description	PCR primer pair^a^
Zero-coverage region	Deletion	*eutB* (SR35_12495) Δ(*intZ-yffS*) *eutA* (SR35_12500)	CPZ-55 prophage exists only in K-12 MG1655	None^b^
		*pepD* (SR35_01230) Δ(*gpt-ykfC*) IS*5 mmuP* (SR35_01240)	CP4–6 prophage (exclusive of IS*5* at the right end) and the upstream *gpt-proA* exist only in K-12 MG1655	
		*quuD* (SR35_02745) SR35_02750 IS*5* Δ(*nmpC-borD*) *ybcV* (SR35_02760)	Within DLP12 prophage region	
		*ttcA* (SR35_06950) Δ(*intR-ynaE*) *uspF* (SR35_06955)	Rac prophage exists only in K-12 MG1655; First eight amino acids of TtcA protein are not identical between RR1 and K-12 MG1655	
		*yjiPQ* (SR35_22365) IS*1* Δ(*yjiV-hpaC*) *hpaB* (SR35_22380)	*mcrBC*, *hsdRMS*, and *mrr* genes all deleted; 3′-end (18 bp) of *hpaB* was truncated (compared with BL21(DE3))	
Breakpoint analysis	Insertion (IS)	*cytR* (SR35_20155) IS*2 priA* (SR35_20170)	IS*2* inserted	P3-P4
		*cutA* (SR35_21255) IS*2 dcuA* (SR35_21270)	IS*2* inserted	P5-P6
		*lacY*::IS*1* (SR35_01670)	IS*1* insertion leads to *lacY1* mutation	P7-P8
		*mglA*::IS*1* (SR35_10960)	IS*1* inserted	P9-P10
		*ybhM* (SR35_03975)::IS*5*	IS*5* inserted at N-terminal part of *ybhM*	P11-P12
		*ydeP* (SR35_07605) IS*5 ydeQ* (SR35_07615)	IS*5* inserted	P13-P14
		*ymdA* (SR35_05320)::IS*1*	IS*1* inserted at C-terminal part of *ydmA*	P15-P16
		*ynfB* (SR35_08035)::IS*2*	IS*2* inserted at N-terminal part of *ynfB*	P17-P18
	Deletion (IS-mediated)	*flhD* (SR35_09665) ΔIS*1 uspC* (SR35_09670)	IS*1* exists only in K-12 MG1655	P19-P20
		*ychE* (SR35_06365) ΔIS*5U oppA* (SR35_06370)	IS*5U* exists only in K-12 MG1655	P21-P22

*If deletions involve more than two genes, internal ones are abbreviated using a hyphen. A double colon after a gene symbol designates an IS insertion. Genes surrounding an IS are shown if the IS was inserted in an intergenic region*.

^a^*Primer pair P1-P2 (not shown here) was used for the amplification of K-12 ‘island’ in the B genomic background (see text)*.

^b^*PCR and Sanger sequencing were not required for the confirmation of zero-coverage regions*.

## Results and Discussion

### The History of the RR1 Strain

**FIGURE 1:**
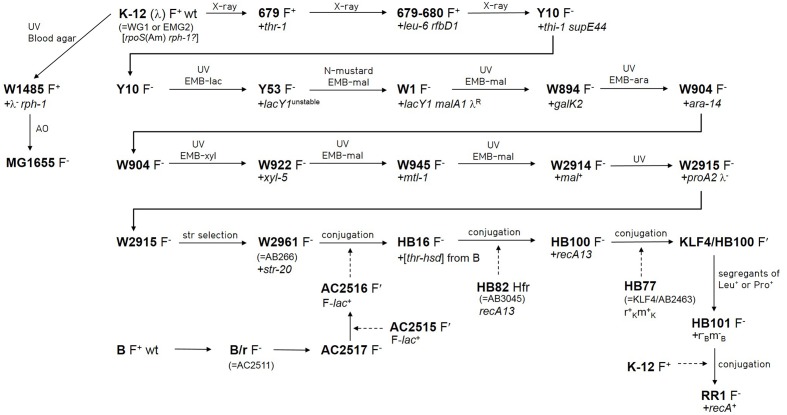
**Pedigree of *Escherichia coli* RR1 and other related strains**. Newly introduced mutations are denoted after a plus sign. Lineage from wild-type *E. coli* K-12 to W2961 was based on Bachmann (1972, 1996). Lineage from W2961 to HB101 was based on Boyer and Roulland-Dussoix (1969), and Rothen (1997). Linage from wild-type K-12 to MG1655 was based on Bachmann (1996) and Hayashi et al. (2006). Lineage from wild-type B to AC2516 was based on Boyer (1964) and Rothen (1997). Conjugal transfer of genes is denoted by dashed arrows. AC2515 is a *Salmonella typhosa* strain carrying F-*lac* from *E. coli* (Johnson et al., 1964). KLF4 is an F′ factor (Low, 1968) that was derived from AB259, an Hfr (Hayes) strain. Note that *rpoS*(Am) and *rph-1* mutations do not appear in Bachmann (1972, 1996). AO and EMB indicate acridine orange and eosin methylene blue agar, respectively.

The genealogy from wild-type *E. coli* K-12 (F^+^ λ ^+^) to HB101, a hybrid strain of *E. coli* K-12 and B, was traced by literature search (**Figure 1**). The pedigrees from wild-type *E. coli* K-12 to Y10 (F^-^ *thr-1 leu-6 thi-1 supE44*) (Lederberg and Tatum, 1946; Lederberg, 1947, 1952; Tatum and Lederberg, 1947), and from Y10 to W2961 [= AB266; F^-^ *araC14 leuB6*(Am) Δ(*gpt-proA*)*62 lacY1 glnX44*(AS) *galK2*(Oc) λ *- Rac-0 rfbC1 mgl-51 rpsL20*(Str^R^) *xylA5 mtl-1 thiE1*] (Sypherd, 1965) are well-documented in Bachmann’s work (Bachmann, 1972, 1996). The earlier auxotrophic mutations up to Y10 were introduced by X-ray irradiation, whereas the mutations in latter generations leading to W2961 were introduced by repetitive UV irradiation and subsequent use of various selective media. The generations from W2961 via HB101 (RecA^-^) to RR1 (RecA^+^) were all produced using F′- or Hfr-mediated conjugation. However, HB101 has been incorrectly described in a literature (Singer and Berg, 1991) as being derived from RR1 by mutation of the *recA* gene.

HB101 is one of the restriction-modification deficient mutants (r^-^_B_ m^-^_B_) produced by the pioneering work of Boyer and Roulland-Dussoix (1969) to investigate the genetic basis of the restriction-modification system. HB101 was the first mutant to indicate the presence of the third cistron (*ramC*, currently known as *hsdS*) that comprises the restriction-modification system. In their study, r^+^_B_ m^+^_B_ alleles and *thr*^+^ from the B strain were simultaneously introduced into the genome of AB266 to make HB16. The *recA13* mutation was introduced into the HB16 strain by conjugation with the HB82 Hfr strain (= AB3045) to make HB100. Subsequently, different r^-^_B_ m^-^_B_ mutants were segregated from a cross between HB100 and HB77.

The entire evolutionary process from AB266 to HB101, including details of all participant strains, was further elaborated by Rothen (1997). For example, while the B donor strain for r^+^_B_ m^+^_B_ alleles was not specified in the original report (Boyer and Roulland-Dussoix, 1969), Rothen reported that AC2517 (B/r F^-^) (Boyer, 1964) was converted to a conjugal donor F′ strain (AC2516) (Boyer, 1964) after crossing with AC2515 (F′-*lac*^+^) (Johnson et al., 1964), and that HB16 was produced by the cross between AC2516 and AB266, not by P1 production. By contrast, Boyer and Roulland-Dussoix stated that r^+^_B_ m^+^_B_ alleles were “co-transduced” with *thr*^+^ genes. The B genomic fragment (190.4 kb; including the ∼20 kb region that was later deleted by IS*1*-mediated recombination; see below) harbored in the genome of RR1 is too large to be introduced by a single phage P1 transduction event. Therefore, the term “co-transduced” seems to be used as a generic term describing the introduction of foreign DNA.

Although the RR1 strain is frequently mentioned in the scientific literature, and even in online lists of laboratory *E. coli* strains, we could not discover for what purpose RR1 was constructed from HB101. Raymond L. Rodriguez explained via personal communication as follows: RR1 was constructed by conjugal mating of HB101 (*recA13*) with an F^+^ RecA^+^ strain with a view to using it as a host for colicin-resistant plasmids that work best in a RecA^+^ background. We found that the genomic position (2,772,443–2,806,787) including *recA* (SR35_13775) in the RR1 strain was identical to the intact form found in K-12 MG1655 (RecA^+^). Sanger sequencing of PCR products amplified from *recA* loci of several strains confirmed that only HB101 strains have a mutant *recA13* allele (Leu^52^→Phe).

### The K-12 and B Hybrid Genome of RR1

**FIGURE 2:**
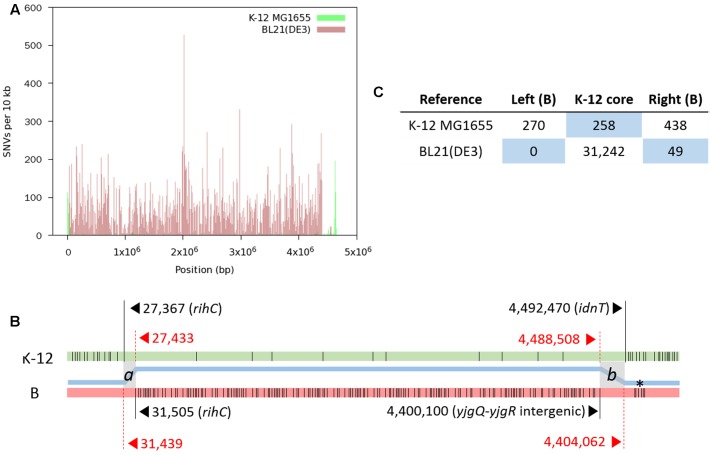
**Distribution of SNVs identified by mapping RR1 reads to reference genomes**. **(A)** SNV density plot across each reference genome. **(B)** A schematic representation of SNVs (thin vertical lines) on each reference genome (not drawn to scale). For each block of SNVs representing the heterologous genomic segments, the first and last SNVs are denoted by long vertical lines with coordinates and gene symbols. The thick blue line between the two genomes depicts the hybrid genome structure resulting from recombination between the K-12 (green) and B (pink) genomes. Two crossover events took place at the gray zones (*a* and *b*), where the two parental genomes are identical to each other. Locus tags of RR1 for *rihC*, *yjgQ*, *yjgR*, and *idnT* are SR35_00165, SR35_21895, SR35_21900, and SR35_21910, respectively. An asterisk denotes the K-12 island in the B region. **(C)** Total numbers of SNVs in each block on the reference genomes. The number of SNVs in the right B-block includes one SNV within the *b* crossover region. The blue background emphasizes recombinational blocks within the parental genomes.

We produced a total of 48,255,170 paired reads (4.97 Gb) from the library with an average insert size of 359 bp. Of these reads, 44,074,332 (4.24 Gb; 92.9% paired) passed quality trimming and filtering, and were used for mapping. SNV distribution shows that the finalized RR1 genome is a hybrid of K-12 (∼95.9%) and B (∼4.1%) (**Figure 2**); these proportions were later revised taking indels into account. A complete list of SNVs identified from the first round of read mapping was given in Supplementary Table S1. Given that B genomic DNA was introduced by a single event, two recombinational junctions where crossover occurred were defined between the last SNV identified in one genome reference and the first SNV in the other reference. In fact, the two junctions are short stretches of nucleotides in which K-12 MG1655 and BL21(DE3) are identical with each other, such that homologous recombination can occur. When K-12 MG1655 was used as the reference, 270 SNVs were densely distributed in a narrow genome range (∼27 kb; 302–27,367), where the last nucleotide position corresponds to 31,439 in the BL21(DE3) genome. On the other hand, when BL21(DE3) was used as the reference, the first SNV occurred at 31,505, corresponding to 27,433 in the K-12 MG1655 genome. When the same approach was applied to the other recombinational junction, we could construct the first hybrid sequence (4,651,433 bp) consisting of 1–31,504 from BL21(DE3), 27,433–4,488,508 from K-12 MG1655, and 4,400,100–4,558,953 from BL21(DE3).

Using this sequence as the starting reference, three rounds of mapping, variant calling, and consensus extraction were performed. During the initial mapping step, five zero-coverage regions that could be due to large-scale deletions were identified from visual inspection of aligned reads (Supplementary Figure S1). With iterative mapping and reference correction, discrepancies between reads and the reference sequence were gradually mitigated and finally, confirmatory read mapping revealed that a correct genome sequence with regard to large deletions could be reconstructed without the need for PCR and Sanger sequencing.

Large-scale insertions, however, could not be accurately reconstructed by mapping reads and revising reference sequence only. Therefore, candidate regions identified by manual inspection of read depth and by breakpoint analysis were amplified by PCR and confirmed by Sanger sequencing. Large-scale insertions and deletions, mostly results of prophage- or insertion sequence (IS)-mediated events, are summarized in **Table 2**. Meanwhile, a moderate length deletion (123 bp) resulting in a truncation at the C-terminus of *yghQ* (SR35_15225) (data not shown) did not appear as a conspicuous zero-coverage region, leaving a stretch of 66 Ns after the final consensus sequence extraction. This region was corrected by manual *in silico* extension of reference sequence using the unaligned ends of partially mapped reads at the position followed by sequence joining.

The length of finalized genome sequence of RR1 is 4,587,291 bp with 50.8% G+C. If recombinational junctions are defined as *a* and *b* (see **Figure 2B**), we can say that 31,349 ≤*a* < 31,505 and 4,447,909 < *b* ≤ 4,451,871 in RR1 genome coordinates, which means the length of extant B genomic DNA introduced by transduction is 166.8–170.9 kb. A single nucleotide C to T variation at 4,448,417 [4,489,016 and 4,400,608 in K-12 MG1655 and BL21(DE3), respectively; within *yjgR* of the right recombinational junction] was identified whichever references were used, implying this mutation occurred after the introduction of the B genomic fragment.

### Reconstruction of the RR1 Hybrid Genome in Detail

During visual investigation of aligned reads on the B reference genome, an SNV-rich segment that was almost identical to K-12 MG1655 (99.97% identical; only 1-bp difference out of 3521 bp) sequence was found at position 4,577,076–4,580,596 and was verified using PCR and Sanger sequencing. The segment corresponded to 4,548,791–4,552,258 in B genome coordinates and contained *slt-trpR-yjjX-ytjC* (*gpmB*)-*rob* (SR35_22630-SR35_22655; Supplementary Figure S2). Out of 48 SNVs occurring in the B genome segment, 45 were concentrated in this narrow 3.5 kb region (Supplementary Table S1). They were ∼136 and ∼38 kb apart from each end of recombinational junctions, respectively. We confirmed the presence of this small K-12 island in all five strains chosen for this study (two RR1 strains and three HB101 strains) using PCR and Sanger sequencing. We also encountered an opposite situation in the K-12 genomic background of RR1 strain, where B-like 17 SNVs were concentrated in a short 2.1-kb region (*yaaU-kefF-kefC*; SR35_00255-SR35_00265; Supplementary Table S1). Because this region is only ∼18 kb apart from the last recombinational junction (*rihC*; SR35_00165), the integration at the secondary site might have occurred concomitantly with the primary integration event. Such dispersed integration was discussed in the previous report (Studier et al., 2009). The presence of flanking sequences (>3 kb both) where K-12 and B genome sequences are identical with each other (Supplementary Figure S3) might have facilitated the integration of B-like “islet” in the K-12 genome region.

Special care had to be taken when resolving the boundaries for invertible P-DNA segment (GenBank X01805.1) (Plasterk and van de Putte, 1985), a 1,797 bp long element flanked by two inverted repeat sequences (5′-TTGGTTTGGGAGAAGG-3′) within the cryptic prophage e14. New “junction sites,” identified by the structural variant detection function of CLC Genomics Workbench and breseq during the validation process of the final genome sequence, suggested that the sequencing library is mixed such that roughly half of the library molecules have inversions. PCR results of junction regions also suggested that the genomic DNA used for sequencing library construction had both orientations of the internal sequence element (data not shown). When reads were mapped with a high-stringency condition (match score 1, mismatch cost 5, length fraction 0.99, and similarity fraction 0.99) simultaneously to the two P-DNA segments in both directions having 1 kb flanking sequences beyond terminal repeats, average read coverage was 460.17 (normal direction in compliance with NC_000913.3) and 511.73 (inverted orientation). Even though the cell culture for genomic DNA preparation was inoculated from a single colony, we observed the dynamic inversion of the P-segment in the single strain population during a short-term period. A BLAST search against the nucleotide collection at NCBI showed that there are at least five genome sequences having a P-DNA segment with inverted orientation besides X01805.1. CLC Genomics Workbench and breseq also suggested a low coverage region at the 3′-end of *rhsA* gene, but deletion was not detected except for correcting two nucleotides using PCR and Sanger sequencing (data not shown).

Most of the genes in the genomic fragment derived from the B strain are syntenic with respect to their K-12 counterparts, but some of them have different amino acid sequences, indicating that they may have functional differences with respect to the parental K-12 strain. The presence or absence of genes specific to each strain confers phenotypical differences. For example, SR35_00100–SR35_00110 encoding fimbrial proteins and type III effector-like protein (between *nhaR* and IS*1*) is present only in B, a choline transporter downstream of the *fec* cluster (SR35_22010) is present only in B, the *yjhIHGFU* cluster is present only in K-12, the *nanCMS* cluster for the utilization of sialic acid is present only in K-12, *fimB* (SR35_22195) is interrupted by IS*1* in B but is intact in K-12, a *yjiV-hpaB* (C-terminal) deletion encompassing the entire *mrr-hsdRMS-mcrBC* locus is present only in RR1, the *hpa* cluster for 4-hydroxyphenylacetic acid catabolite pathway is present only in B, and *yjjJ* is present only in K-12.

The immigration control region (ICR), consisting of the *mrr-hsdRMS-mcrBC* gene cluster, together with the O-antigen region, is the most divergent region between *E. coli* strains (Milkman et al., 2003). The *hpa* cluster is downstream of the ICR. Because the ICR and neighboring genes are completely absent in the RR1 genome (see below), the KpLE2 prophage-like region (SR35_21940–SR35_22160) in the B genomic fragment is the most divergent compared with its cognate K-12 region. The aforementioned choline transporter gene and *yjhIHGFU* cluster are all located in the KpLE2 prophage region. The presence of ISs was one of the main factors contributing to inter-strain sequence variation in this prophage region. In K-12 MG1655, there are single copies of IS*2*, IS*4*, IS*911* (interrupted by the following IS*30*), IS*30* and IS*1*, but RR1 has only IS*911* (uninterrupted; SR35_22030) and IS*1* (SR35_22080-SR35_22090) surrounding *fecIRABCDE* operon.

### Genome-Based Elucidation of RR1 Genotype

The complete genome sequence of RR1 can help us to elucidate all known and yet-to-be-discovered characteristics of RR1 (**Table 3**), but also tell us more about its history. We should be aware that genomic differences between RR1 and K-12 MG1655 are in fact the sum of mutations occurring in each descendant from the common ancestor, wild-type *E. coli* K-12 (Bachmann, 1972, 1996). Strain MG1655 (F^-^ λ ^-^ *rph-1*) was rapidly obtained from wild-type K-12 via W1485 (F^+^ λ ^-^ *rph-1*) after only one round of UV irradiation and acridine orange mutagenesis (Bachmann, 1996). Compared to genomic differences between *E. coli* B strains REL606 and BL21(DE3) (Jeong et al., 2009), most of which were caused by disparate integration of K-12 DNA in a narrow region of the genome (Studier et al., 2009), the 258 SNVs between RR1 and MG1655 constitute a much larger difference in the same K-12 genomic background. The extensive mutagenesis by X-ray and UV radiation of the RR1 lineage (**Figure 1**) might account for this observation.

**Table 3:** Genotypes and characteristics of *Escherichia coli* HB101 and RR1 strains reported in the literature or referenced on websites.

Characteristic description^a^	Reference or website
**HB101**	
F^-^ Pro^-^ Gal^-^ Str^R^ Rec^-^ r^-^_B_ m^-^_B_	Boyer and Roulland-Dussoix, 1969
F^-^ *hsdS20*(r^-^_B_ m^-^_B_) *recA13 ara-14 proA*2 *lacY1 galK2 rpsL20*(Sm^r^) *xyl-5 mtl-1 supE44* λ ^-^	Maniatis et al., 1982
F^-^ *araC14 leuB6*(Am) Δ(*gpt-proA*)*62 lacY1 glnX44*(AS) *galK2*(Oc) λ ^-^ *recA13 rpsL20*(strR) *xylA5 mtl-1 thiE1* [*hsdS20*]	The Coli Genetics Stock Center^b^
F^-^ *mcrB mrr hsdS20*(r^-^_B_ m^-^_B_) *recA13 leuB6 ara-14 proA*2 *lacY1 galK2 xyl-5 mtl-1 rpsL20*(Str^R^) *glnV44* λ ^-^	OpenWetWare^c^
F^-^ Δ(*gpt-proA*)*62 leuB6 glnV44 ara-14 galK2 lacY1*Δ(*mcrC-mrr*) *rpsL20*(Str^R^) *xyl-5 mtl-1 recA13 thi-1*	NEB^d^
F^-^ Δ(*gpt-proA*)*62 leuB6 glnV44 ara-14 galK2 lacY1 Δ*(*mcrC-mrr*) *rpsL20*(Str^R^) *xyl-5 mtl-1 recA13*	Sigma-Aldrich^e^
**RR1**	
F^-^ *pro leu thi lacY Str*^R^ r^-^_K_ m^-^_K_	Bolivar et al., 1977
F^-^; the same as HB101 except *recA^+^*	Maniatis et al., 1982
HB101 *recA*^+^	OpenWetWare
HB101 RecA^+^	Sigma-Aldrich

^a^*Instead of using the standardized nomenclatures of genotypes and phenotypes, original descriptions from references were followed*.

^b^*http://cgsc.biology.yale.edu/;*

^c^*http://openwetware.org/;*

^d^*https://www.neb.com/;*

^e^*http://www.sigmaaldrich.com/.*

As shown in **Table 3**, the characteristics of RR1 and HB101 strains reported in the literature or on websites show some discrepancies. For example, Bolivar et al. (1977) mistakenly described RR1 as r^-^_K_ m^-^_K_, whereas its parental strain HB101 had been clearly described as r^-^_B_ m^-^_B_. The most significant discrepancy concerns Δ(*mcrC-mrr*) or the *mcrC mrr* genotype. Our sequencing results demonstrated an IS-mediated deletion (∼20 kb) from the *yjiV* gene at the 3′-end of the *hpaB* gene (Supplementary Figure S1). The deleted region includes *mrr-hsdRMS-mcrBC*, whose deletion results in more permissive host strain than that of strains harboring point mutations (Woodcock et al., 1989; Grant et al., 1990; Doherty et al., 1991). We assumed that the *mcrC-mrr* deletion leading to the r^-^_B_ m^-^_B_ phenotype did not occur when HB101 was made from HB100 (r^+^_B_ m^+^_B_). Reportedly, HB101 is *trans* dominant to the r^+^ phenotype, as shown by complementation analysis (Boyer and Roulland-Dussoix, 1969), which means that the original HB101 strain produces mutant HsdS proteins (caused by *hsdS20* mutation) that interfere with wild-type restriction function (Arber and Linn, 1969). Therefore, our sequencing results showed that IS-mediated deletion of *mrr-hsdRMS-mcrBC* must have occurred in a later generation of the original HB101 cells harboring the *hsdS20* mutation. We checked the prevalence of the *mrr-hsdRMS-mcrBC* deletion in the five HB101 strains using PCR and Sanger sequencing. All tested HB101 strains harbored this deletion, implying that it might have occurred immediately after the construction of HB101 and might not be confined to the RR1 lineage.

### Ancient Mutations Revisited

The genotype of wild-type K-12, which comprises most of the RR1 genome, differs slightly from source to source. Bachmann (1972, 1996) stated that wild-type K-12 is simply F^+^ λ ^+^ in the pedigree figures omitting *rfb-50* or Δ*rfb-51* mutations common to K-12 wild-types, but mentioned these mutations in the main text of references. Hayashi et al. (2006) stated that, without mentioning *rfb* mutations, wild-type *E. coli* K-12 has *rpoS*(Am) and *rph-1* (frameshift) mutations, while K-12 MG1655 has the pseudorevertant allele (Q33) at the *rpoS* locus. However, in Bachmann’s pedigree, the *rph-1* mutation appears in W1485 for the first time, not in its parent wild-type K-12. The genotype of K-12 MG1655 (F^-^ λ ^-^ *ilvG*^-^ *rfb-50 rph-1*) is in good agreement with current genome sequence information of K-12 MG1655. The genotype inconsistency implies either that mutations found later have not yet been added to the list of wild-type characteristics, or that different culture stocks of the same strain have independent variations (Freddolino et al., 2012). Alternatively, it may arise from misinterpretation of experimental data or literature search results.

Notably, the *rfbD1* mutation in the dTDP-4-dehydrorhamnose reductase gene is absent from currently available genotypes of HB101, RR1, Bachmann’s first pedigrees of K-12, and their mutant derivatives (Bachmann, 1972). However, later work (Bachmann, 1996) stated that *rfbD1* is present in Y10 and its direct descendants. We found a frameshift mutation in the *rfbD* gene (SR35_10405) in the genome of the RR1 strain, which is identical to that found in recently sequenced *E. coli* ER1821R (Jobling et al., 2016), a K-12 derivative laboratory strain harboring the ancestral *rfbD1* mutation. An IS*5* insertion (SR35_10355), designated *rfb-50*, that is common in K-12 wild-type strains, was also found in the RR1 strain. Our complete genome sequence can explain the genotype or mutations of the RR1 strain (**Table 4**).

**Table 4:** Genotype of the RR1 strain based on its complete genome sequence.

Genotype	Mutations revealed by comparisons with wild-type gene sequences
*araC14* (= *ara-14*)	Ser^262^ → Pro (SR35_00345)
Δ(*gpt-proA*)*62* (= *proA2*)	Δ(*gpt-ykfC*), IS-mediated deletion
*galK2*	Glu^134^ → STOP (SR35_03810)
*glnX44* (= *glnV44, supE44*)	The presence of tRNA-Gln(CUG) (SR35_03325)
*hsdS20*	Incompatible with *mcrB mrr*; because *hsdS* gene does not exist in Δ(*mrr-hsdRMS-mcrBC*) background
*lacY1*	IS*1* insertion (SR35_01670)
*leuB6* (= *leu-6*)	Ser^286^ → Leu (SR35_00395)
*mlc*^a^	Gln^369^ → STOP (SR35_08090)
*mtl-1*	Multiple mutations in *mtlA* (SR35_18425)
*recA*^+^	Wild-type *recA* (SR35_13775)
*rfb-50*	IS*5* insertion (SR35_10355) at the downstream end or *rfb* operon
*rfbD1*^a^	Frameshift (SR35_10405)
*rpoS*(Am)^a^	Gln^33^ → STOP (SR35_13990)
*rpsL20* (= *str-20*)	Lys^43^ → Thr (SR35_17055)
*thiE1* (= *thi-1*)	Asp^70^ → Ala (SR35_20475)
*xylA5* (= *xyl-5*)	Trp^69^ → STOP (SR35_18240)

^a^*Newly identified by genome sequencing*.

Whereas an amber mutation in *rpoS* was found in RR1 (SR35_13990), no mutation in the *rph* gene was observed. This implies that the *rph-1* mutation might have occurred in the descendant of wild-type K-12, contrary to a previous report (Hayashi et al., 2006). Because Jensen (1993) observed the *rph* mutation only in MG1655, W3110, and their common ancestor W1485, the original wild-type strain may have a normal *rph* allele. Unexpectedly, we observed an amber mutation (C1105T) in the open reading frame of the *mlc* gene (also known as *dgsA*) in the RR1 strain, which encodes a global regulator of carbohydrate uptake including the glucose phosphotransferase system. Although it is not known how the RR1 strain obtained the amber mutation, DNA sequencing of the *mlc* gene of HB101 strains revealed the same mutation, indicating that disruption of the *mlc* gene occurred before the construction of the RR1 strain.

## Conclusion

For several decades, *E. coli* laboratory strains have undergone adaptation and evolution as a result of X-ray or UV irradiation, as well as by recombining foreign DNA into their genomes, which can also occur in nature. Precise genomic sequences of descendant strains reveal predicted and unexpected mutations that can be used to interpret the trajectories of genome evolution and the physiology of each strain.

## Author Contributions

HJ and SJL designed the research; HJ, YMS, and HJK performed genome sequencing; HJ and SJL analyzed the data; and HJ and SJL wrote the paper.

## Conflict of Interest Statement

The authors declare that the research was conducted in the absence of any commercial or financial relationships that could be construed as a potential conflict of interest.

## Footnotes

1 http://www.phrap.org/
