# Supplementary material for: Unveiling the Hybrid Genome Structure of Escherichia coli RR1 (HB101 RecA+)
Source: Front Microbiol. 2017 Apr 4;8:585. doi: 10.3389/fmicb.2017.00585 (PMC5379014; doi:10.3389/fmicb.2017.00585)
Supplement: Supplementary file 1 [file Data_Sheet_1.DOCX]

Supplementary Material

Unveiling the Hybrid Genome Structure of *Escherichia coli* RR1 (HB101 RecA+)

**Haeyoung Jeong*, Young Mi Sim, Hyun Ju Kim, Sang Jun Lee***

*** Correspondence: Haeyoung Jeong** [**hyjeong@kribb.re.kr**](mailto:hyjeong@kribb.re.kr) **or Sang Jun Lee** [**sangjlee@cau.ac.kr**](mailto:sangjlee@cau.ac.kr)


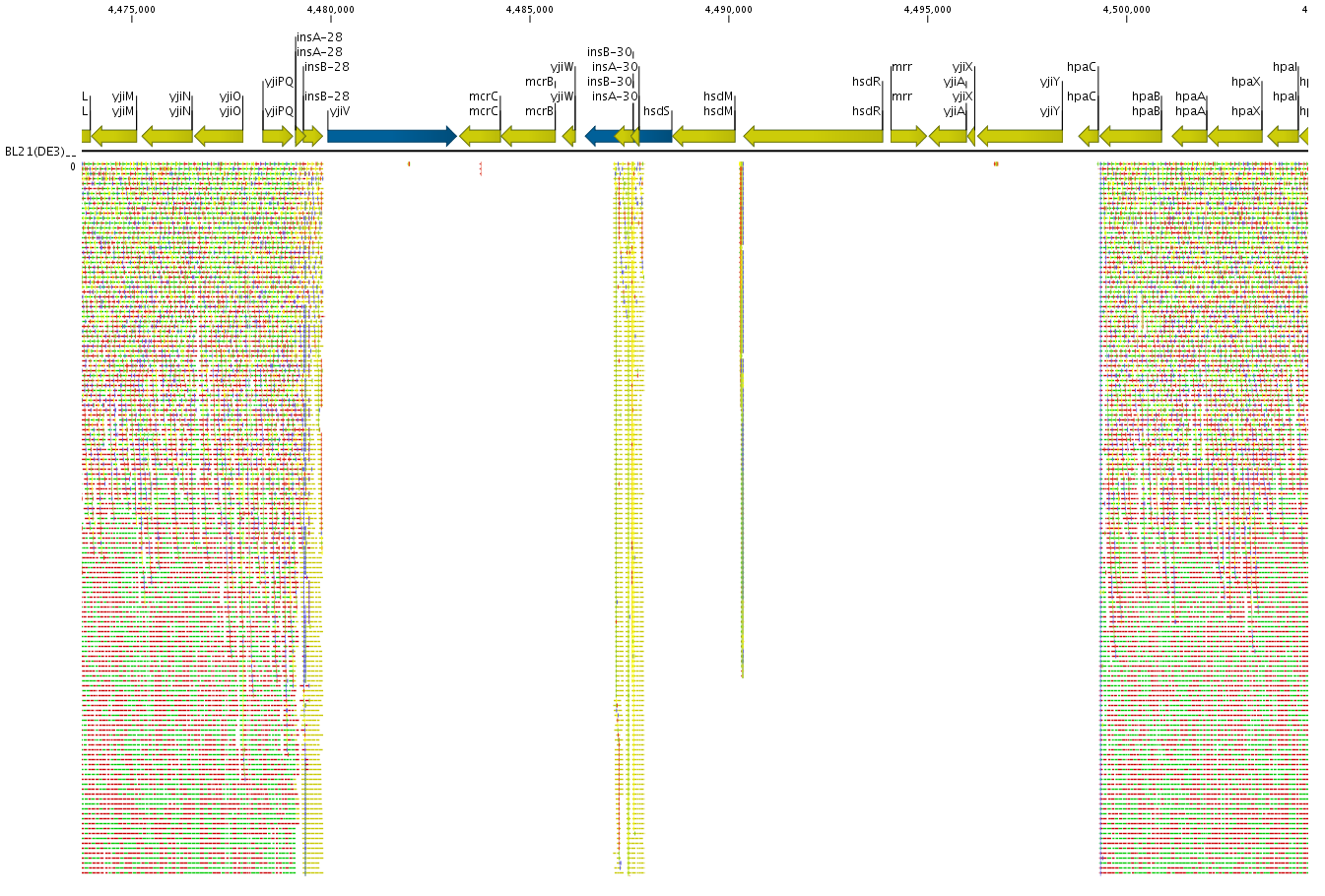


## Supplementary Figure S1. Large deletion of genomic region surrounding the *mrr-hsdRMS-mcrBC* locus in RR1 genome. A ~20-kb zero-coverage region shown by read mapping on BL21(DE3) reference under CLC Genomics Workbench environment.


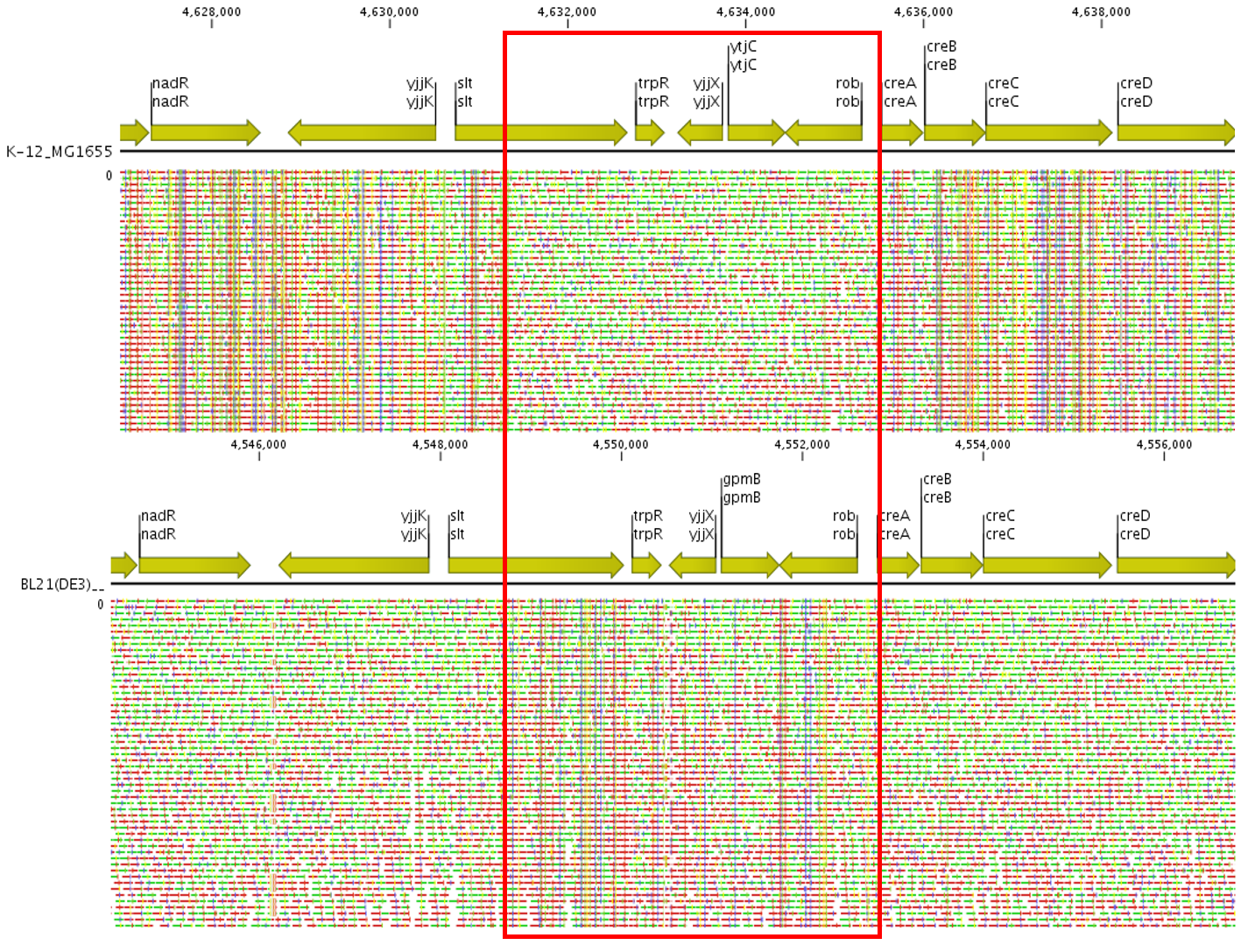


## Supplementary Figure S2. K-12 ‘island’ within B genomic background. The K-12 (~3.5 kb) island (indicated by a red box) was identified by read mapping to K-12 MG1655 genome (top) and BL21(DE3) genome (bottom), which were used as references. Vertical lines throughout aligned reads represents variants.

##


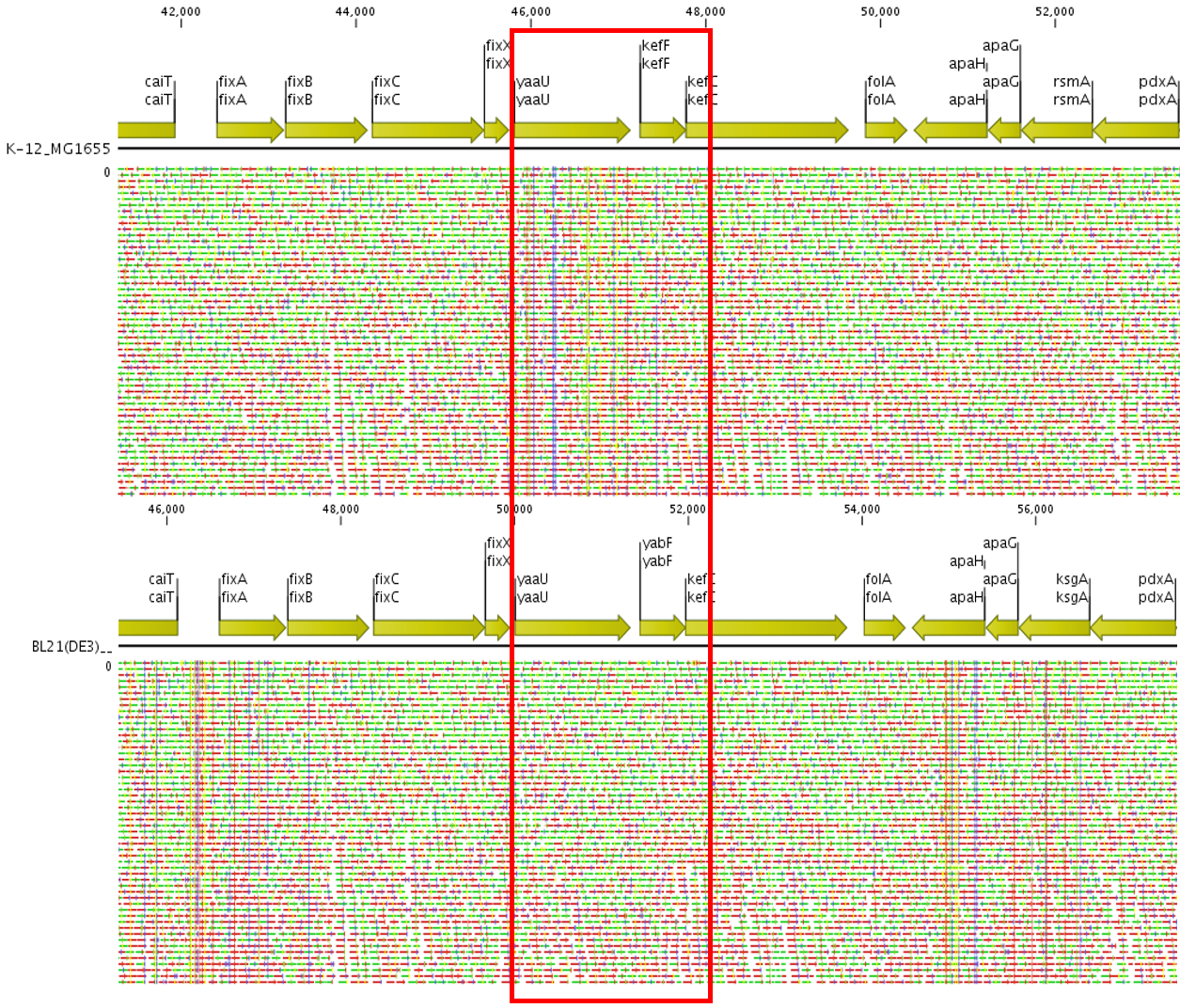


## Supplementary Figure S3. B-like ‘islet’ within K-12 genomic background. The B-like islet (indicated by a red box) was identified by read mapping to K-12 MG1655 genome (top) and BL21(DE3) genome (bottom), which were used as references. Vertical lines throughout aligned reads represents variants. Note that there are no variations within the sequences surrounding the red box (at least 3 kb to both directions).

## Supplementary Table S1. SNVs in RR1 genome with respect to cognate reference genomes. The genomes of K-12 MG1655 and BL21(DE3) were used as cognate references to analyze true SNVs. A variation found in the *b* crossover region is indicated in red. The K-12 island within the B genomic region is colored in green. B-like islet in K-12 genomic region is colored in blue. The original variant table in Excel format, including additional columns such as count and coverage values, are available at <http://genoglobe.kr/kribb/escherichia_coli_rr1>.

| **Reference** | **Ref. Position** | **Type** | **Size** | **Ref.**  **base** | **Var. base** | **Overlapping annotations** | **Coding region change** | **Amino acid change** |
| --- | --- | --- | --- | --- | --- | --- | --- | --- |
| K-12 | 45950 | SNV | 1 | G | A | Gene: yaaU, CDS: yaaU | NP_414587.1:c.144G>A |  |
| K-12 | 45978 | SNV | 1 | C | T | Gene: yaaU, CDS: yaaU | NP_414587.1:c.172C>T |  |
| K-12 | 46028 | SNV | 1 | T | C | Gene: yaaU, CDS: yaaU | NP_414587.1:c.222T>C |  |
| K-12 | 46247 | SNV | 1 | T | C | Gene: yaaU, CDS: yaaU | NP_414587.1:c.441T>C |  |
| K-12 | 46253 | SNV | 1 | G | C | Gene: yaaU, CDS: yaaU | NP_414587.1:c.447G>C |  |
| K-12 | 46277 | SNV | 1 | T | C | Gene: yaaU, CDS: yaaU | NP_414587.1:c.471T>C |  |
| K-12 | 46448 | SNV | 1 | C | A | Gene: yaaU, CDS: yaaU | NP_414587.1:c.642C>A |  |
| K-12 | 46634 | SNV | 1 | G | T | Gene: yaaU, CDS: yaaU | NP_414587.1:c.828G>T |  |
| K-12 | 46652 | SNV | 1 | A | G | Gene: yaaU, CDS: yaaU | NP_414587.1:c.846A>G |  |
| K-12 | 46655 | SNV | 1 | A | G | Gene: yaaU, CDS: yaaU | NP_414587.1:c.849A>G |  |
| K-12 | 46661 | SNV | 1 | T | C | Gene: yaaU, CDS: yaaU | NP_414587.1:c.855T>C |  |
| K-12 | 46790 | SNV | 1 | A | G | Gene: yaaU, CDS: yaaU | NP_414587.1:c.984A>G |  |
| K-12 | 46943 | SNV | 1 | T | C | Gene: yaaU, CDS: yaaU | NP_414587.1:c.1137T>C |  |
| K-12 | 46976 | SNV | 1 | A | G | Gene: yaaU, CDS: yaaU | NP_414587.1:c.1170A>G |  |
| K-12 | 47099 | SNV | 1 | G | A | Gene: yaaU, CDS: yaaU | NP_414587.1:c.1293G>A |  |
| K-12 | 47434 | SNV | 1 | T | C | Gene: kefF, CDS: kefF | NP_414588.1:c.189T>C |  |
| K-12 | 48033 | SNV | 1 | T | G | Gene: kefC, CDS: kefC | NP_414589.1:c.265T>G | NP_414589.1:p.Cys89Gly |
| K-12 | 66635 | SNV | 1 | C | T | Repeat region: REP6 element |  |  |
| K-12 | 71170 | SNV | 1 | T | C | Gene: araC, CDS: araC | NP_414606.1:c.784T>C | NP_414606.1:p.Ser262Pro |
| K-12 | 81102 | SNV | 1 | G | A | Gene: leuB, CDS: leuB | NP_414615.4:c.857C>T | NP_414615.4:p.Ser286Leu |
| K-12 | 154182 | SNV | 1 | C | T | Gene: htrE, CDS: htrE | NP_414681.1:c.1245G>A |  |
| K-12 | 156068 | SNV | 1 | A | G | Gene: yadV, CDS: yadV | NP_414682.1:c.134T>C | NP_414682.1:p.Val45Ala |
| K-12 | 271817 | SNV | 1 | G | A | Gene: insN, CDS: insN (prophage CP4-6) | insN:c.312G>A |  |
| K-12 | 271822 | SNV | 1 | G | A | Gene: insN, CDS: insN (prophage CP4-6) | insN:c.317G>A | insN:p.Arg106His |
| K-12 | 271834 | SNV | 1 | C | T | Gene: insN, CDS: insN (prophage CP4-6) | insN:c.329C>T | insN:p.Ala110Val |
| K-12 | 271838 | SNV | 1 | T | A | Gene: insN, CDS: insN (prophage CP4-6) | insN:c.333T>A |  |
| K-12 | 271876 | SNV | 1 | A | G | Gene: insN, CDS: insN (prophage CP4-6) | insN:c.371A>G | insN:p.Lys124Arg |
| K-12 | 271901 | SNV | 1 | G | A | Gene: insN, CDS: insN (prophage CP4-6) | insN:c.396G>A |  |
| K-12 | 284535 | SNV | 1 | T | G | Gene: yagF, CDS: yagF | NP_414803.1:c.1335T>G |  |
| K-12 | 292019 | SNV | 1 | C | T | Gene: yagJ, CDS: yagJ (prophage CP4-6) | yagJ:c.616C>T |  |
| K-12 | 309381 | SNV | 1 | C | T | Gene: ecpB, CDS: ecpB | NP_414826.1:c.646G>A | NP_414826.1:p.Asp216Asn |
| K-12 | 309985 | SNV | 1 | T | A | Gene: ecpB, CDS: ecpB | NP_414826.1:c.42A>T |  |
| K-12 | 321260 | SNV | 1 | A | T |  |  |  |
| K-12 | 341180 | SNV | 1 | C | T | Gene: yahJ, CDS: yahJ | NP_414858.1:c.56C>T | NP_414858.1:p.Thr19Ile |
| K-12 | 410666 | SNV | 1 | C | T | Gene: mak, CDS: mak | NP_414928.2:c.523C>T | NP_414928.2:p.Pro175Ser |
| K-12 | 421027 | SNV | 1 | C | T | Gene: proY, CDS: proY | NP_414936.1:c.42C>T |  |
| K-12 | 472849 | SNV | 1 | A | G | Gene: glnK, CDS: glnK | NP_414984.1:c.252A>G |  |
| K-12 | 495622 | SNV | 1 | C | T | Gene: htpG, CDS: htpG | NP_415006.1:c.503C>T | NP_415006.1:p.Thr168Ile |
| K-12 | 500988 | SNV | 1 | C | T | Gene: gsk, CDS: gsk | NP_415010.1:c.864C>T |  |
| K-12 | 514434 | SNV | 1 | C | T | Gene: ybbJ, CDS: ybbJ | NP_415021.2:c.426G>A |  |
| K-12 | 534742 | SNV | 1 | G | A | Gene: gcl, CDS: gcl | NP_415040.1:c.827G>A | NP_415040.1:p.Gly276Asp |
| K-12 | 582597 | SNV | 1 | C | T | Gene: ybcY, CDS: ybcY (prophage DLP12) | ybcY:c.212G>A | ybcY:p.Ser71Asn |
| K-12 | 602835 | SNV | 1 | G | A | Gene: pheP, CDS: pheP | NP_415108.1:c.877G>A | NP_415108.1:p.Ala293Thr |
| K-12 | 674259 | SNV | 1 | G | A | Gene: leuS, CDS: leuS | NP_415175.1:c.525C>T |  |
| K-12 | 677829 | SNV | 1 | T | C | Gene: djlB, CDS: djlB | NP_415179.1:c.415T>C | NP_415179.1:p.Trp139Arg |
| K-12 | 694941 | SNV | 1 | G | A | Gene: miaB, CDS: miaB | NP_415194.1:c.15C>T |  |
| K-12 | 696470 | SNV | 1 | C | T | Gene: glnX, tRNA: glnX |  |  |
| K-12 | 708774 | SNV | 1 | G | A | Gene: chiP, CDS: chiP | NP_415207.1:c.441G>A |  |
| K-12 | 721604 | SNV | 1 | G | A | Gene: kdpE, CDS: kdpE | NP_415222.1:c.130C>T | NP_415222.1:p.Arg44Cys |
| K-12 | 780624 | SNV | 1 | C | T | Gene: lysT, tRNA: lysT |  |  |
| K-12 | 789580 | SNV | 1 | C | A | Gene: galK, CDS: galK | NP_415278.1:c.400G>T | NP_415278.1:p.Glu134* |
| K-12 | 822527 | SNV | 1 | C | T | Gene: clsB, CDS: clsB | NP_415310.1:c.1213G>A | NP_415310.1:p.Glu405Lys |
| K-12 | 883396 | SNV | 1 | C | T |  |  |  |
| K-12 | 908169 | SNV | 1 | C | T | Gene: ybjT, CDS: ybjT | NP_415390.4:c.114G>A |  |
| K-12 | 968252 | SNV | 1 | G | A | Gene: msbA, CDS: msbA | NP_415434.1:c.1632G>A |  |
| K-12 | 1054855 | SNV | 1 | G | A | Gene: torS, CDS: torS | NP_415513.2:c.1324C>T | NP_415513.2:p.Gln442* |
| K-12 | 1066699 | SNV | 1 | C | T | Gene: agp, CDS: agp | NP_415522.1:c.1115C>T | NP_415522.1:p.Ala372Val |
| K-12 | 1075234 | SNV | 1 | C | T | Gene: putA, CDS: putA | NP_415534.1:c.3649G>A | NP_415534.1:p.Val1217Ile |
| K-12 | 1075528 | SNV | 1 | C | T | Gene: putA, CDS: putA | NP_415534.1:c.3355G>A | NP_415534.1:p.Ala1119Thr |
| K-12 | 1078268 | SNV | 1 | A | T | Gene: putA, CDS: putA | NP_415534.1:c.615T>A | NP_415534.1:p.Phe205Leu |
| K-12 | 1086153 | SNV | 1 | C | T | Gene: pgaD, CDS: pgaD | NP_415540.1:c.367G>A | NP_415540.1:p.Gly123Arg |
| K-12 | 1090508 | SNV | 1 | C | T | Gene: pgaA, CDS: pgaA | NP_415543.1:c.1782G>A |  |
| K-12 | 1107209 | SNV | 1 | C | T | Gene: clsC, CDS: clsC | NP_415564.2:c.855C>T |  |
| K-12 | 1109740 | SNV | 1 | C | T | Gene: opgG, CDS: opgG | NP_415566.1:c.406C>T | NP_415566.1:p.Leu136Phe |
| K-12 | 1110033 | SNV | 1 | G | A | Gene: opgG, CDS: opgG | NP_415566.1:c.699G>A |  |
| K-12 | 1112209 | SNV | 1 | C | T | Gene: opgH, CDS: opgH | NP_415567.1:c.1347C>T |  |
| K-12 | 1112421 | SNV | 1 | C | T | Gene: opgH, CDS: opgH | NP_415567.1:c.1559C>T | NP_415567.1:p.Ser520Phe |
| K-12 | 1114389 | SNV | 1 | C | T | Gene: mdtG, CDS: mdtG | NP_415571.1:c.1102G>A | NP_415571.1:p.Ala368Thr |
| K-12 | 1115185 | SNV | 1 | C | T | Gene: mdtG, CDS: mdtG | NP_415571.1:c.306G>A |  |
| K-12 | 1117707 | SNV | 1 | G | A | Gene: yceA, CDS: yceA | NP_415573.1:c.901G>A | NP_415573.1:p.Gly301Ser |
| K-12 | 1120111 | SNV | 1 | G | A | Gene: solA, CDS: solA | NP_415577.1:c.476C>T | NP_415577.1:p.Ala159Val |
| K-12 | 1121547 | SNV | 1 | G | A |  |  |  |
| K-12 | 1121762 | SNV | 1 | G | A | Gene: pyrC, CDS: pyrC | NP_415580.1:c.846C>T |  |
| K-12 | 1123823 | MNV | 2 | GT | AG | Gene: grxB, CDS: grxB | NP_415582.1:c.231_232delACinsCT | NP_415582.1:p.Leu77_Leu78delinsPheLeu |
| K-12 | 1126316 | SNV | 1 | G | A | Gene: yceH, CDS: yceH | NP_415585.1:c.160G>A | NP_415585.1:p.Glu54Lys |
| K-12 | 1126518 | SNV | 1 | C | T | Gene: yceH, CDS: yceH | NP_415585.1:c.362C>T | NP_415585.1:p.Ala121Val |
| K-12 | 1127334 | SNV | 1 | G | A | Gene: yceM, CDS: yceM | NP_415586.1:c.529G>A | NP_415586.1:p.Gly177Ser |
| K-12 | 1127338 | SNV | 1 | G | A | Gene: yceM, CDS: yceM | NP_415586.1:c.533G>A | NP_415586.1:p.Gly178Asp |
| K-12 | 1129796 | SNV | 1 | G | A | Gene: flgN, CDS: flgN | NP_415588.1:c.35C>T | NP_415588.1:p.Ser12Phe |
| K-12 | 1130210 | SNV | 1 | G | A | Gene: flgA, CDS: flgA | NP_415590.1:c.654C>T |  |
| K-12 | 1130803 | SNV | 1 | C | T | Gene: flgA, CDS: flgA | NP_415590.1:c.61G>A | NP_415590.1:p.Ala21Thr |
| K-12 | 1133273 | SNV | 1 | G | A | Gene: flgE, CDS: flgE | NP_415594.1:c.700G>A | NP_415594.1:p.Gly234Ser |
| K-12 | 1134466 | SNV | 1 | C | T | Gene: flgF, CDS: flgF | NP_415595.1:c.665C>T | NP_415595.1:p.Ala222Val |
| K-12 | 1138152 | SNV | 1 | C | T | Gene: flgJ, CDS: flgJ | NP_415599.1:c.782C>T | NP_415599.1:p.Thr261Ile |
| K-12 | 1138533 | SNV | 1 | C | T | Gene: flgK, CDS: flgK | NP_415600.1:c.156C>T |  |
| K-12 | 1138831 | SNV | 1 | C | T | Gene: flgK, CDS: flgK | NP_415600.1:c.454C>T | NP_415600.1:p.Arg152Cys |
| K-12 | 1141865 | SNV | 1 | G | A | Gene: rne, CDS: rne | NP_415602.1:c.2503C>T | NP_415602.1:p.Pro835Ser |
| K-12 | 1144974 | SNV | 1 | C | T | Gene: rluC, CDS: rluC | NP_415604.1:c.35C>T | NP_415604.1:p.Ala12Val |
| K-12 | 1146110 | SNV | 1 | C | T | Gene: yceF, CDS: yceF | NP_415605.2:c.486G>A |  |
| K-12 | 1146384 | SNV | 1 | A | G | Gene: yceF, CDS: yceF | NP_415605.2:c.212T>C | NP_415605.2:p.Val71Ala |
| K-12 | 1147792 | SNV | 1 | T | A | Gene: plsX, CDS: plsX | NP_415608.2:c.172T>A | NP_415608.2:p.Ser58Thr |
| K-12 | 1161403 | SNV | 1 | G | A | Gene: fhuE, CDS: fhuE | NP_415620.1:c.149C>T | NP_415620.1:p.Ala50Val |
| K-12 | 1169836 | SNV | 1 | A | G | Gene: ycfS, CDS: ycfS | NP_415631.1:c.539T>C | NP_415631.1:p.Leu180Pro |
| K-12 | 1171417 | SNV | 1 | G | A | Gene: mfd, CDS: mfd | NP_415632.1:c.2548C>T |  |
| K-12 | 1176602 | SNV | 1 | C | T | Gene: lolC, CDS: lolC | NP_415634.1:c.1176C>T |  |
| K-12 | 1180126 | SNV | 1 | G | A | Gene: cobB, CDS: cobB | NP_415638.2:c.496G>A | NP_415638.2:p.Ala166Thr |
| K-12 | 1181995 | SNV | 1 | G | A | Gene: potD, CDS: potD | NP_415641.1:c.835C>T |  |
| K-12 | 1189980 | SNV | 1 | A | G | Gene: phoP, CDS: phoP | NP_415648.1:c.468T>C |  |
| K-12 | 1193141 | SNV | 1 | G | A | Gene: mnmA, CDS: mnmA | NP_415651.4:c.633C>T |  |
| K-12 | 1194922 | SNV | 1 | G | A | Gene: rluE, CDS: rluE | NP_415653.4:c.30C>T |  |
| K-12 | 1201548 | SNV | 1 | A | C | Gene: ymfI, CDS: ymfI (prophage e14) | NP_415661.2:c.52A>C | NP_415661.2:p.Thr18Pro |
| K-12 | 1201550 | SNV | 1 | C | A | Gene: ymfI, CDS: ymfI (prophage e14) | NP_415661.2:c.54C>A |  |
| K-12 | 1227656 | SNV | 1 | G | A |  |  |  |
| K-12 | 1244908 | SNV | 1 | C | T | Gene: ymgE, CDS: ymgE | NP_415713.1:c.181C>T |  |
| K-12 | 1252059 | SNV | 1 | C | T | Gene: dhaR, CDS: dhaR | NP_415719.2:c.994C>T | NP_415719.2:p.Pro332Ser |
| K-12 | 1252663 | SNV | 1 | C | T | Gene: dhaR, CDS: dhaR | NP_415719.2:c.1598C>T | NP_415719.2:p.Ser533Phe |
| K-12 | 1253002 | SNV | 1 | C | T |  |  |  |
| K-12 | 1282857 | SNV | 1 | C | T | Gene: narG, CDS: narG | NP_415742.1:c.2994C>T |  |
| K-12 | 1306736 | SNV | 1 | T | G | Gene: oppF, CDS: oppF | NP_415763.1:c.973T>G | NP_415763.1:p.Ser325Ala |
| K-12 | 1308684 | SNV | 1 | T | C |  |  |  |
| K-12 | 1323798 | SNV | 1 | G | A | Gene: yciV, CDS: yciV | NP_415782.1:c.579G>A |  |
| K-12 | 1325054 | SNV | 1 | G | A | Gene: yciQ, CDS: yciQ | NP_415784.1:c.309G>A | NP_415784.1:p.Trp103* |
| K-12 | 1331059 | MNV | 2 | TC | AT | Gene: topA, CDS: topA | NP_415790.1:c.12_13delTCinsAT | NP_415790.1:p.Ala4_Leu5delinsAlaPhe |
| K-12 | 1337394 | SNV | 1 | A | G | Gene: acnA, CDS: acnA | NP_415792.1:c.1564A>G | NP_415792.1:p.Ser522Gly |
| K-12 | 1341542 | SNV | 1 | G | A | Gene: yciM, CDS: yciM | NP_415796.1:c.985G>A | NP_415796.1:p.Ala329Thr |
| K-12 | 1358859 | SNV | 1 | T | C | Gene: puuP, CDS: puuP | NP_415812.4:c.329A>G | NP_415812.4:p.Tyr110Cys |
| K-12 | 1375737 | SNV | 1 | G | A | Gene: ycjQ, CDS: ycjQ | NP_415829.1:c.775G>A | NP_415829.1:p.Ala259Thr |
| K-12 | 1405758 | SNV | 1 | G | A |  |  |  |
| K-12 | 1490095 | SNV | 1 | G | A | Gene: gapC, CDS: gapC | gapC:c.619C>T | gapC:p.His207Tyr |
| K-12 | 1629247 | SNV | 1 | C | T | Gene: ydfZ, CDS: ydfZ | NP_416059.1:c.33C>T |  |
| K-12 | 1632513 | SNV | 1 | C | T | Misc. feature: cryptic prophage Qin/Kim | ydfJ:c.85-228G>A |  |
| K-12 | 1643679 | SNV | 1 | A | T | Gene: ydfU, CDS: ydfU (prophage Qin/Kim) | ydfJ:c.[84+9076T>A]; NP_416078.4:c.[626T>A] | NP_416078.4:p.Leu209Gln |
| K-12 | 1652331 | SNV | 1 | T | C | Gene: intQ, CDS: intQ (prophage Qin/Kim) | ydfJ:c.[84+424A>G]; intQ:c.[781T>C] | intQ:p.Phe261Leu |
| K-12 | 1660102 | SNV | 1 | C | T | Gene: ynfE, CDS: ynfE | NP_416104.1:c.2034C>T |  |
| K-12 | 1667460 | SNV | 1 | G | A | Gene: mlc, CDS: mlc | NP_416111.1:c.1105C>T | NP_416111.1:p.Gln369* |
| K-12 | 1894839 | SNV | 1 | T | C | Gene: pabB, CDS: pabB | NP_416326.1:c.35T>C | NP_416326.1:p.Leu12Pro |
| K-12 | 2040433 | SNV | 1 | C | A | Gene: yedY, CDS: yedY | NP_416480.1:c.956C>A | NP_416480.1:p.Ala319Asp |
| K-12 | 2111256 | Deletion | 1 | G | - | Gene: rfbD, CDS: rfbD | NP_416544.1:c.721delC | NP_416544.1:p.Leu241fs |
| K-12 | 2173361 | Deletion | 2 | CC | - | Gene: gatC | gatC:c.914+3_914+4delGG |  |
| K-12 | 2173448 | SNV | 1 | C | T | Gene: gatC, CDS: gatC | gatC:c.831G>A |  |
| K-12 | 2204395 | SNV | 1 | T | A |  |  |  |
| K-12 | 2348366 | SNV | 1 | C | A | Gene: nrdB, CDS: nrdB | NP_416738.1:c.983C>A | NP_416738.1:p.Thr328Lys |
| K-12 | 2388753 | SNV | 1 | G | A | Gene: yfbO, CDS: yfbO | NP_416777.2:c.119G>A | NP_416777.2:p.Gly40Asp |
| K-12 | 2512423 | MNV | 2 | CA | TT | Gene: mntH, CDS: mntH | NP_416893.1:c.283_284delTGinsAA | NP_416893.1:p.Trp95Lys |
| K-12 | 2577937 | SNV | 1 | T | C | Gene: maeB, CDS: maeB | NP_416958.1:c.441A>G |  |
| K-12 | 2582272 | SNV | 1 | C | T | Gene: ypfG, CDS: ypfG | NP_416961.1:c.506G>A | NP_416961.1:p.Gly169Glu |
| K-12 | 2587112 | SNV | 1 | C | T | Gene: narQ, CDS: narQ | NP_416964.1:c.1382C>T | NP_416964.1:p.Ala461Val |
| K-12 | 2600978 | SNV | 1 | C | T |  |  |  |
| K-12 | 2613763 | SNV | 1 | G | A | Gene: hyfR, CDS: hyfR | NP_416986.4:c.1864G>A | NP_416986.4:p.Glu622Lys |
| K-12 | 2623838 | SNV | 1 | G | A | Gene: ppk, CDS: ppk | NP_416996.1:c.795G>A |  |
| K-12 | 2632711 | SNV | 1 | G | A | Gene: guaB, CDS: guaB | NP_417003.1:c.1360C>T | NP_417003.1:p.Arg454Cys |
| K-12 | 2684686 | SNV | 1 | A | C | Gene: glyA, CDS: glyA | NP_417046.1:c.822T>G |  |
| K-12 | 2722946 | SNV | 1 | G | A | Gene: pssA, CDS: pssA | NP_417080.4:c.220G>A | NP_417080.4:p.Ala74Thr |
| K-12 | 2774947 | SNV | 1 | G | A | Gene: yfjW, CDS: yfjW (prophage CP4-57) | NP_417129.1:c.1630G>A | NP_417129.1:p.Gly544Ser |
| K-12 | 2778119 | SNV | 1 | C | T |  |  |  |
| K-12 | 2806897 | SNV | 1 | C | A | Gene: proW, CDS: proW | NP_417164.1:c.888C>A |  |
| K-12 | 2814595 | SNV | 1 | C | T | Gene: luxS, CDS: luxS | NP_417172.1:c.139G>A | NP_417172.1:p.Val47Met |
| K-12 | 2849941 | SNV | 1 | G | A |  |  |  |
| K-12 | 2851821 | SNV | 1 | G | A | Gene: hypB, CDS: hypB | NP_417207.1:c.821G>A | NP_417207.1:p.Gly274Asp |
| K-12 | 2867455 | SNV | 1 | G | A | Gene: rpoS, CDS: rpoS | NP_417221.1:c.97C>T | NP_417221.1:p.Gln33* |
| K-12 | 2880635 | SNV | 1 | T | C | Gene: casD, CDS: casD | NP_417237.2:c.414A>G |  |
| K-12 | 2881212 | SNV | 1 | C | T | Gene: casC, CDS: casC | NP_417238.1:c.931G>A | NP_417238.1:p.Ala311Thr |
| K-12 | 2883115 | SNV | 1 | G | A | Gene: casA, CDS: casA | NP_417240.1:c.1024C>T | NP_417240.1:p.Pro342Ser |
| K-12 | 2886731 | SNV | 1 | T | C | Gene: ygcB, CDS: ygcB | NP_417241.1:c.489A>G | NP_417241.1:p.Ile163Met |
| K-12 | 2890626 | SNV | 1 | G | A | Gene: cysJ, CDS: cysJ | NP_417244.1:c.1273C>T | NP_417244.1:p.Leu425Phe |
| K-12 | 2893219 | SNV | 1 | C | T | Gene: ygcN, CDS: ygcN | NP_417246.4:c.563C>T | NP_417246.4:p.Ser188Phe |
| K-12 | 2893647 | SNV | 1 | C | T | Gene: ygcN, CDS: ygcN | NP_417246.4:c.991C>T | NP_417246.4:p.Gln331* |
| K-12 | 2894839 | SNV | 1 | C | T | Repeat region: REP201 element |  |  |
| K-12 | 2916216 | SNV | 1 | G | A | Gene: barA, CDS: barA | NP_417266.1:c.1160G>A | NP_417266.1:p.Gly387Glu |
| K-12 | 2926772 | SNV | 1 | C | A | Gene: ygdH, CDS: ygdH | NP_417275.1:c.465C>A |  |
| K-12 | 2934092 | SNV | 1 | G | A |  |  |  |
| K-12 | 3025597 | Deletion | 1 | T | - | Gene: xanQ, CDS: xanQ | NP_417358.2:c.1247delT | NP_417358.2:p.Ile416fs |
| K-12 | 3070858 | SNV | 1 | T | G | Gene: fbaA, CDS: fbaA | NP_417400.1:c.387A>C | NP_417400.1:p.Lys129Asn |
| K-12 | 3096754 | SNV | 1 | G | A | Gene: rdgB, CDS: rdgB | NP_417429.1:c.74G>A | NP_417429.1:p.Gly25Asp |
| K-12 | 3207548 | Deletion | 1 | G | - | Gene: ttdB, CDS: ttdB | NP_417534.1:c.178delG | NP_417534.1:p.Gly60fs |
| K-12 | 3256301 | SNV | 1 | G | A | Gene: yhaM, CDS: yhaM | YP_026202.1:c.351C>T |  |
| K-12 | 3279276 | SNV | 1 | C | G | Gene: kbaZ, CDS: kbaZ | NP_417601.1:c.363C>G | NP_417601.1:p.Phe121Leu |
| K-12 | 3296481 | SNV | 1 | G | A | Gene: yraP, CDS: yraP | NP_417619.1:c.73G>A | NP_417619.1:p.Val25Met |
| K-12 | 3329743 | SNV | 1 | G | T | Gene: dacB, CDS: dac | NP_417649.1:c.781G>T | NP_417649.1:p.Asp261Tyr |
| K-12 | 3364664 | SNV | 1 | G | A | Gene: yhcD, CDS: yhcD | NP_417683.1:c.1858G>A | NP_417683.1:p.Gly620Arg |
| K-12 | 3388041 | SNV | 1 | T | G | Gene: aaeB, CDS: aaeB | NP_417707.1:c.148A>C | NP_417707.1:p.Thr50Pro |
| K-12 | 3411689 | SNV | 1 | G | T | Gene: yhdJ, CDS: yhdJ | NP_417728.4:c.37G>T | NP_417728.4:p.Glu13* |
| K-12 | 3433665 | SNV | 1 | C | T |  |  |  |
| K-12 | 3433900 | SNV | 1 | C | T | Gene: def, CDS: def | NP_417745.1:c.211C>T |  |
| K-12 | 3474425 | SNV | 1 | T | G | Gene: rpsL, CDS: rps | NP_417801.1:c.128A>C | NP_417801.1:p.Lys43Thr |
| K-12 | 3481131 | SNV | 1 | G | A | Gene: kefG, CDS: kefG | NP_417810.1:c.31C>T |  |
| K-12 | 3486502 | SNV | 1 | C | T | Gene: crp, CDS: crp | NP_417816.1:c.383C>T | NP_417816.1:p.Thr128Ile |
| K-12 | 3500129 | SNV | 1 | G | A | Gene: frlA, CDS: frlA | NP_417829.2:c.220G>A | NP_417829.2:p.Gly74Ser |
| K-12 | 3511466 | SNV | 1 | C | T | Gene: yhfZ, CDS: yhfZ | YP_026214.1:c.879G>A |  |
| K-12 | 3517171 | SNV | 1 | G | A | Gene: damX, CDS: damX | NP_417847.1:c.136C>T | NP_417847.1:p.Leu46Phe |
| K-12 | 3555047 | MNV | 2 | GT | TG | Gene: malT, CDS: mal | NP_417877.1:c.1963_1964delGTinsTG | NP_417877.1:p.Val655Cys |
| K-12 | 3560362 | SNV | 1 | G | A | Gene: glpR, CDS: glpR | glpR:c.242C>T | glpR:p.Ala81Val |
| K-12 | 3560456 | Insertion | 1 | - | G | Gene: glpR, CDS: glpR | glpR:c.150_150+1insC |  |
| K-12 | 3591074 | SNV | 1 | G | A | Gene: ugpB, CDS: ugpB | NP_417910.1:c.1252C>T |  |
| K-12 | 3630221 | SNV | 1 | C | T | Gene: yhiI, CDS: yhiI | NP_417944.1:c.382G>A | NP_417944.1:p.Glu128Lys |
| K-12 | 3646970 | SNV | 1 | G | T | Gene: gor, CDS: gor | NP_417957.1:c.672G>T | NP_417957.1:p.Gln224His |
| K-12 | 3647428 | SNV | 1 | C | T | Gene: gor, CDS: gor | NP_417957.1:c.1130C>T | NP_417957.1:p.Ala377Val |
| K-12 | 3647949 | SNV | 1 | C | T |  |  |  |
| K-12 | 3664594 | SNV | 1 | G | A | Gene: gadW, CDS: gadW | NP_417972.1:c.25C>T | NP_417972.1:p.Leu9Phe |
| K-12 | 3665576 | SNV | 1 | C | T | Gene: gadX, CDS: gadX | NP_417973.1:c.235G>A | NP_417973.1:p.Val79Ile |
| K-12 | 3707883 | SNV | 1 | G | A |  |  |  |
| K-12 | 3707947 | SNV | 1 | C | A |  |  |  |
| K-12 | 3719429 | SNV | 1 | G | A |  |  |  |
| K-12 | 3725176 | SNV | 1 | T | G | Gene: glyQ, CDS: glyQ | NP_418017.1:c.143A>C | NP_418017.1:p.Glu48Ala |
| K-12 | 3729440 | MNV | 2 | CC | TT |  |  |  |
| K-12 | 3730560 | SNV | 1 | C | T | Gene: xylA, CDS: xylA | NP_418022.1:c.206G>A | NP_418022.1:p.Trp69* |
| K-12 | 3770539 | SNV | 1 | C | T | Gene: yibH, CDS: yibH | NP_418054.1:c.841G>A | NP_418054.1:p.Val281Met |
| K-12 | 3773171 | Deletion | 1 | G | - | Gene: mtlA, CDS: mtlA | NP_418056.1:c.891delG | NP_418056.1:p.Pro297fs |
| K-12 | 3773176 | SNV | 1 | C | T | Gene: mtlA, CDS: mtlA | NP_418056.1:c.896C>T | NP_418056.1:p.Ser299Phe |
| K-12 | 3815880 | Insertion | 1 | - | C | Gene: rph, CDS: rph | rph:c.671_672insG | rph:p.Gly224fs |
| K-12 | 3834564 | SNV | 1 | G | A | Gene: yicJ, CDS: yicJ | NP_418114.4:c.1366C>T | NP_418114.4:p.Gln456* |
| K-12 | 3926991 | SNV | 1 | G | A | Gene: asnC, CDS: asnC | NP_418199.1:c.13C>T |  |
| K-12 | 3931779 | SNV | 1 | T | C | Gene: kup, CDS: kup | YP_026244.1:c.464T>C | YP_026244.1:p.Phe155Ser |
| K-12 | 3966530 | MNV | 2 | CC | TT | Gene: rho, CDS: rho | NP_418230.1:c.114_115delCCinsTT |  |
| K-12 | 3967770 | SNV | 1 | C | T |  |  |  |
| K-12 | 4133252 | SNV | 1 | C | T | Gene: metF, CDS: metF | NP_418376.1:c.637C>T | NP_418376.1:p.Pro213Ser |
| K-12 | 4193996 | SNV | 1 | T | G | Gene: thiE, CDS: thiE | NP_418421.1:c.209A>C | NP_418421.1:p.Asp70Ala |
| K-12 | 4215670 | SNV | 1 | G | T | Gene: aceB, CDS: aceB | NP_418438.1:c.193G>T | NP_418438.1:p.Asp65Tyr |
| K-12 | 4223638 | SNV | 1 | T | C |  |  |  |
| K-12 | 4238232 | SNV | 1 | C | T | Gene: yjbH, CDS: yjbH | NP_418453.1:c.599C>T | NP_418453.1:p.Pro200Leu |
| K-12 | 4272711 | SNV | 1 | C | T | Gene: uvrA, CDS: uvrA | NP_418482.1:c.1161G>A |  |
| K-12 | 4275204 | SNV | 1 | C | T |  |  |  |
| K-12 | 4276212 | SNV | 1 | C | T | Gene: yjcC, CDS: yjcC | NP_418485.1:c.742C>T | NP_418485.1:p.Pro248Ser |
| K-12 | 4276422 | SNV | 1 | C | T | Gene: yjcC, CDS: yjcC | NP_418485.1:c.952C>T | NP_418485.1:p.Pro318Ser |
| K-12 | 4281093 | SNV | 1 | G | A | Gene: yjcE, CDS: yjcE | NP_418489.1:c.1114G>A | NP_418489.1:p.Ala372Thr |
| K-12 | 4283043 | SNV | 1 | G | A | Gene: yjcF, CDS: yjcF | NP_418490.1:c.33C>T |  |
| K-12 | 4283465 | SNV | 1 | G | A | Gene: actP, CDS: actP | NP_418491.1:c.1438C>T | NP_418491.1:p.Pro480Ser |
| K-12 | 4284646 | SNV | 1 | G | A | Gene: actP, CDS: actP | NP_418491.1:c.257C>T | NP_418491.1:p.Ala86Val |
| K-12 | 4285238 | SNV | 1 | G | A |  |  |  |
| K-12 | 4288385 | SNV | 1 | G | A | Gene: nrfA, CDS: nrfA | NP_418494.1:c.622G>A | NP_418494.1:p.Val208Ile |
| K-12 | 4296381 | Insertion | 2 | - | CG | Repeat region: RIP321 element |  |  |
| K-12 | 4296428 | SNV | 1 | G | A | Repeat region: RIP321 element |  |  |
| K-12 | 4297782 | SNV | 1 | G | A | Gene: fdhF, CDS: fdhF | NP_418503.1:c.1585C>T | NP_418503.1:p.Pro529Ser |
| K-12 | 4298401 | SNV | 1 | G | A | Gene: fdhF, CDS: fdhF | NP_418503.1:c.966C>T |  |
| K-12 | 4306035 | SNV | 1 | C | T | Gene: yjcS, CDS: yjcS | NP_418507.2:c.563G>A | NP_418507.2:p.Gly188Asp |
| K-12 | 4306960 | SNV | 1 | G | A | Gene: alsK, CDS: alsK | NP_418508.1:c.840C>T |  |
| K-12 | 4307151 | SNV | 1 | G | A | Gene: alsK, CDS: alsK | NP_418508.1:c.649C>T | NP_418508.1:p.Pro217Ser |
| K-12 | 4318215 | SNV | 1 | G | A | Gene: phnK, CDS: phnK | YP_026282.1:c.550C>T |  |
| K-12 | 4320007 | SNV | 1 | G | A | Gene: phnI, CDS: phnI | NP_418523.1:c.657C>T |  |
| K-12 | 4320344 | SNV | 1 | G | A | Gene: phnI, CDS: phnI | NP_418523.1:c.320C>T | NP_418523.1:p.Ala107Val |
| K-12 | 4320416 | SNV | 1 | G | A | Gene: phnI, CDS: phn | NP_418523.1:c.248C>T | NP_418523.1:p.Thr83Ile |
| K-12 | 4320543 | SNV | 1 | G | A | Gene: phnI, CDS: phnI | NP_418523.1:c.121C>T | NP_418523.1:p.Leu41Phe |
| K-12 | 4321488 | SNV | 1 | G | A | Gene: phnG, CDS: phnG | NP_418525.1:c.209C>T | NP_418525.1:p.Ala70Val |
| K-12 | 4321603 | SNV | 1 | T | C | Gene: phnG, CDS: phnG | NP_418525.1:c.94A>G | NP_418525.1:p.Ile32Val |
| K-12 | 4322786 | SNV | 1 | G | A | Gene: phnE, CDS: phnE | phnE:c.455C>T | phnE:p.Thr152Ile |
| K-12 | 4325644 | SNV | 1 | C | T | Gene: yjdN, CDS: yjdN | NP_418531.1:c.98G>A | NP_418531.1:p.Ser33Asn |
| K-12 | 4328622 | SNV | 1 | T | C | Gene: yjdA, CDS: yjdA | NP_418533.1:c.1488T>C |  |
| K-12 | 4331890 | SNV | 1 | C | T | Gene: proP, CDS: proP | NP_418535.1:c.1389C>T |  |
| K-12 | 4340953 | SNV | 1 | C | T | Gene: melR, CDS: melR | NP_418542.1:c.676G>A | NP_418542.1:p.Gly226Arg |
| K-12 | 4344501 | SNV | 1 | G | A | Gene: melB, CDS: melB | NP_418544.2:c.1133G>A | NP_418544.2:p.Gly378Asp |
| K-12 | 4344935 | SNV | 1 | G | A | Gene: yjdF, CDS: yjdF | NP_418545.1:c.624C>T |  |
| K-12 | 4390331 | SNV | 1 | C | T | Gene: psd, CDS: psd | NP_418584.1:c.30G>A |  |
| K-12 | 4402938 | SNV | 1 | G | A | Gene: hflK, CDS: hflK | NP_418595.1:c.901G>A | NP_418595.1:p.Ala301Thr |
| K-12 | 4410859 | SNV | 1 | C | T | Gene: yjfJ, CDS: yjfJ | NP_418603.1:c.307C>T | NP_418603.1:p.Leu103Phe |
| K-12 | 4414192 | SNV | 1 | G | A |  |  |  |
| K-12 | 4430584 | SNV | 1 | C | T | Gene: cycA, CDS: cycA | NP_418629.1:c.721C>T | NP_418629.1:p.Pro241Ser |
| K-12 | 4431220 | SNV | 1 | C | T | Gene: cycA, CDS: cycA | NP_418629.1:c.1357C>T |  |
| K-12 | 4437890 | SNV | 1 | C | T | Gene: ytfI, CDS: ytfI | YP_026285.1:c.184C>T | YP_026285.1:p.Gln62* |
| K-12 | 4439578 | SNV | 1 | G | A |  |  |  |
| K-12 | 4440768 | SNV | 1 | G | A | Gene: ytfL, CDS: ytfL | NP_418639.1:c.448C>T |  |
| K-12 | 4445315 | SNV | 1 | C | T | Gene: tamB, CDS: tam | NP_418642.1:c.1204C>T | NP_418642.1:p.Pro402Ser |
| K-12 | 4445924 | SNV | 1 | C | T | Gene: tamB, CDS: tam | NP_418642.1:c.1813C>T | NP_418642.1:p.Gln605* |
| K-12 | 4446482 | SNV | 1 | C | T | Gene: tamB, CDS: tam | NP_418642.1:c.2371C>T |  |
| K-12 | 4448304 | SNV | 1 | C | T |  |  |  |
| K-12 | 4463952 | SNV | 1 | G | A | Gene: treC, CDS: treC | NP_418660.1:c.758C>T | NP_418660.1:p.Ser253Phe |
| K-12 | 4473900 | SNV | 1 | C | T | Gene: bdcA, CDS: bdcA | NP_418670.1:c.154G>A | NP_418670.1:p.Gly52Arg |
| K-12 | 4475493 | SNV | 1 | T | C | Gene: yjgL, CDS: yjgL | NP_418674.2:c.57T>C |  |
| K-12 | 4476605 | SNV | 1 | G | A | Gene: yjgL, CDS: yjgL | NP_418674.2:c.1169G>A | NP_418674.2:p.Ser390Asn |
| "*b*" region | 4489016 | SNV | 1 | C | T | Gene: yjgR, CDS: yjgR | NP_418684.1:c.1048G>A | NP_418684.1:p.Glu350Lys |
| BL21(DE3) | 4400608 | SNV | 1 | C | T | Gene: yjgR, CDS: yjgR | YP_003056688.1:c.1048G>A | YP_003056688.1:p.Glu350Lys |
| BL21(DE3) | 4520464 | SNV | 1 | C | T | Gene: yjjP, CDS: yjjP | YP_003056792.1:c.770G>A |  |
| BL21(DE3) | 4548791 | SNV | 1 | C | T | Gene: slt, CDS: slt | YP_003056819.1:c.705C>T |  |
| BL21(DE3) | 4548794 | SNV | 1 | A | C | Gene: slt, CDS: slt | YP_003056819.1:c.708A>C |  |
| BL21(DE3) | 4548860 | SNV | 1 | A | G | Gene: slt, CDS: slt | YP_003056819.1:c.774A>G |  |
| BL21(DE3) | 4549098 | SNV | 1 | T | C | Gene: slt, CDS: slt | YP_003056819.1:c.1012T>C |  |
| BL21(DE3) | 4549103 | SNV | 1 | G | T | Gene: slt, CDS: slt | YP_003056819.1:c.1017G>T |  |
| BL21(DE3) | 4549109 | SNV | 1 | G | A | Gene: slt, CDS: slt | YP_003056819.1:c.1023G>A |  |
| BL21(DE3) | 4549152 | SNV | 1 | C | T | Gene: slt, CDS: slt | YP_003056819.1:c.1066C>T |  |
| BL21(DE3) | 4549235 | SNV | 1 | T | A | Gene: slt, CDS: slt | YP_003056819.1:c.1149T>A |  |
| BL21(DE3) | 4549314 | SNV | 1 | T | C | Gene: slt, CDS: slt | YP_003056819.1:c.1228T>C | YP_003056819.1:p.Ser410Pro |
| BL21(DE3) | 4549361 | SNV | 1 | C | T | Gene: slt, CDS: slt | YP_003056819.1:c.1275C>T |  |
| BL21(DE3) | 4549544 | SNV | 1 | T | C | Gene: slt, CDS: slt | YP_003056819.1:c.1458T>C |  |
| BL21(DE3) | 4549553 | SNV | 1 | T | C | Gene: slt, CDS: slt | YP_003056819.1:c.1467T>C |  |
| BL21(DE3) | 4549556 | SNV | 1 | G | T | Gene: slt, CDS: slt | YP_003056819.1:c.1470G>T |  |
| BL21(DE3) | 4549592 | SNV | 1 | C | T | Gene: slt, CDS: slt | YP_003056819.1:c.1506C>T |  |
| BL21(DE3) | 4549616 | SNV | 1 | A | G | Gene: slt, CDS: slt | YP_003056819.1:c.1530A>G |  |
| BL21(DE3) | 4549647 | SNV | 1 | C | T | Gene: slt, CDS: slt | YP_003056819.1:c.1561C>T |  |
| BL21(DE3) | 4549703 | SNV | 1 | T | C | Gene: slt, CDS: slt | YP_003056819.1:c.1617T>C |  |
| BL21(DE3) | 4549715 | SNV | 1 | C | T | Gene: slt, CDS: slt | YP_003056819.1:c.1629C>T |  |
| BL21(DE3) | 4549766 | SNV | 1 | T | C | Gene: slt, CDS: slt | YP_003056819.1:c.1680T>C |  |
| BL21(DE3) | 4549799 | SNV | 1 | C | T | Gene: slt, CDS: slt | YP_003056819.1:c.1713C>T |  |
| BL21(DE3) | 4549907 | SNV | 1 | G | A | Gene: slt, CDS: slt | YP_003056819.1:c.1821G>A |  |
| BL21(DE3) | 4549922 | SNV | 1 | T | C | Gene: slt, CDS: slt | YP_003056819.1:c.1836T>C |  |
| BL21(DE3) | 4549931 | SNV | 1 | A | G | Gene: slt, CDS: slt | YP_003056819.1:c.1845A>G |  |
| BL21(DE3) | 4550030 | SNV | 1 | C | A |  |  |  |
| BL21(DE3) | 4550344 | SNV | 1 | G | A | Gene: trpR, CDS: trpR | YP_003056820.1:c.231G>A |  |
| BL21(DE3) | 4550395 | SNV | 1 | T | C | Gene: trpR, CDS: trpR | YP_003056820.1:c.282T>C |  |
| BL21(DE3) | 4550462 | SNV | 1 | C | T |  |  |  |
| BL21(DE3) | 4550465 | Deletion | 6 | CAGCGT | - |  |  |  |
| BL21(DE3) | 4550540 | SNV | 1 | T | C | Gene: yjjX, CDS: yjjX | YP_003056821.1:c.507A>G |  |
| BL21(DE3) | 4550549 | SNV | 1 | G | A | Gene: yjjX, CDS: yjjX | YP_003056821.1:c.498C>T |  |
| BL21(DE3) | 4550696 | SNV | 1 | G | A | Gene: yjjX, CDS: yjjX | YP_003056821.1:c.351C>T |  |
| BL21(DE3) | 4550867 | SNV | 1 | T | G | Gene: yjjX, CDS: yjjX | YP_003056821.1:c.180A>C |  |
| BL21(DE3) | 4550915 | SNV | 1 | T | C | Gene: yjjX, CDS: yjjX | YP_003056821.1:c.132A>G |  |
| BL21(DE3) | 4551250 | SNV | 1 | T | C | Gene: gpmB, CDS: gpmB | YP_003056822.1:c.153T>C |  |
| BL21(DE3) | 4551748 | SNV | 1 | G | A | Gene: rob, CDS: rob | YP_003056823.1:c.864C>T |  |
| BL21(DE3) | 4551754 | SNV | 1 | A | C | Gene: rob, CDS: rob | YP_003056823.1:c.858T>G |  |
| BL21(DE3) | 4551775 | SNV | 1 | C | T | Gene: rob, CDS: rob | YP_003056823.1:c.837G>A |  |
| BL21(DE3) | 4551805 | SNV | 1 | G | A | Gene: rob, CDS: rob | YP_003056823.1:c.807C>T |  |
| BL21(DE3) | 4552027 | SNV | 1 | T | C | Gene: rob, CDS: rob | YP_003056823.1:c.585A>G |  |
| BL21(DE3) | 4552033 | SNV | 1 | T | C | Gene: rob, CDS: rob | YP_003056823.1:c.579A>G |  |
| BL21(DE3) | 4552084 | SNV | 1 | T | C | Gene: rob, CDS: rob | YP_003056823.1:c.528A>G |  |
| BL21(DE3) | 4552183 | SNV | 1 | C | T | Gene: rob, CDS: rob | YP_003056823.1:c.429G>A |  |
| BL21(DE3) | 4552228 | SNV | 1 | A | G | Gene: rob, CDS: rob | YP_003056823.1:c.384T>C |  |
| BL21(DE3) | 4552255 | SNV | 1 | A | G | Gene: rob, CDS: rob | YP_003056823.1:c.357T>C |  |
| BL21(DE3) | 4552258 | SNV | 1 | C | T | Gene: rob, CDS: rob | YP_003056823.1:c.354G>A |  |
| BL21(DE3) | 4552723 | SNV | 1 | T | G |  |  |  |
| BL21(DE3) | 4553598 | SNV | 1 | C | T | Gene: creB, CDS: creB | YP_003056825.1:c.291C>T |  |

## 
